# Surgery vs. radiotherapy for locally advanced hypopharyngeal cancer in the contemporary era: A population‐based study

**DOI:** 10.1002/cam4.1811

**Published:** 2018-11-26

**Authors:** Yi‐Jun Kim, Rena Lee

**Affiliations:** ^1^ Center for Precision Medicine Seoul National University Hospital Seoul Korea; ^2^ Ewha Womans University Graduate School of Medicine Seoul Korea; ^3^ Department of Radiation Oncology Ewha Womans University College of Medicine Seoul Korea

**Keywords:** chemoradiotherapy, locally advanced hypopharyngeal cancer, SEER, total pharyngectomy

## Abstract

**Objectives:**

To compare overall survival (OS) in locally advanced hypopharyngeal cancer treated with surgery or definitive chemoradiotherapy in the contemporary era.

**Methods:**

From 2010 to 2015, data for patients diagnosed with hypopharyngeal cancer (T2‐T4aM0) and treated with total pharyngectomy with lymph node dissection (surgery group) or definitive radiotherapy and chemotherapy (chemoradiotherapy group) was retrieved from the SEER database. Multivariate analyses were performed in each subgroup divided according to T category (T2‐3 or T4a).

**Results:**

The number of patients in the surgery and chemoradiotherapy groups was 209 and 648, respectively. Among them, the number of T4a patients was 111 and 126 in each group. Three‐year OS rate in the surgery and chemoradiotherapy groups was 37.9% and 44.1%, respectively (*P* = 0.178). The 3‐year OS rate for the T2‐3 patients was 46.5% and 48.7% (*P* = 0.598), and the 3‐year OS rate for the T4a patients was 29.9% and 26.1% in the surgery and chemoradiotherapy groups, respectively (*P* = 0.439). On multivariate analysis, the chemoradiotherapy group was not inferior to the surgery group in T2‐T4a patients (Hazard ratio [HR] for the chemoradiotherapy group 0.889, 95% confidence interval [CI] 0.699‐1.129, *P* = 0.334), in T2‐3 patients (HR 0.932, 95% CI 0.699‐1.297, *P* = 0.675), and in T4a patients (HR 0.880, 95% CI 0.617‐1.256, *P* = 0.481).

**Conclusions:**

Chemoradiotherapy for locally advanced hypophagyngeal cancer showed a comparable OS rate to surgery. For patients with T4a category cancer with high possibility of preserving the laryngopharyngeal function, chemoradiotherapy may be a promising alternative treatment.

## 1 INTRODUCTION

Hypopharyngeal cancer is a poor prognostic cancer.^1^ The 5‐year overall survival (OS) rate is approximately 35%.^2^ Although the survival rate has been significantly improved,^3^ the absolute survival rate has been strikingly restricted for decades compared to human papilloma virus‐related oropharyngeal cancer.^4^

Optimal treatment for locally advanced hypopharyngeal cancer is controversial, especially in T4a cancer. For T4a hypopharyngeal cancer, (chemo)radiotherapy showed a poor survival rate compared to the radical surgery,^2^, ^5^, ^6^ and the invasion of thyroid or cricoid cartilage were considered as to be difficult to preserve laryngopharyngeal function after chemoradiotherapy.^7^ Therefore, surgery is considered as the treatment of choice for T4a cancer. According to the National Comprehensive Cancer Network Categories of Evidence and Consensus, surgery is category 2A (uniform consensus) for T4a hypopharyngeal cancer, whereas chemoradiotherapy is category 3 (major disagreement).^8^ The EHNS‐ESMO‐ESTRO guideline suggests that patients who have massive larynx cartilage invasion are not suitable for organ‐preserving treatment.^9^

However, the use of chemoradiotherapy is increasing and have been the major treatments for locally advanced hypopharyngeal cancer.^10^ Recently, techniques of radiotherapy have been improved. Intensity‐modulated radiotherapy (IMRT) has been gradually generalized in clinic since early 2000,^11^ and IMRT and accelerated radiotherapy showed an improved local control rate compared to conventional 3‐dimensional conformal radiotherapy.^12^, ^13^ Combined chemotherapy also showed improved treatment outcomes.^14^ Therefore, the latest treatment outcomes of chemoradiotherapy for locally advanced hypopharyngeal cancer including T4a cancer might be improved compared to the historical reports.

In this study, we analyzed the latest population‐based database to compare chemoradiotherapy with surgery in locally advanced pharyngeal cancer.

## 2 METHODS AND MATERIALS

### 2.1 Patient population

**Figure 1:**
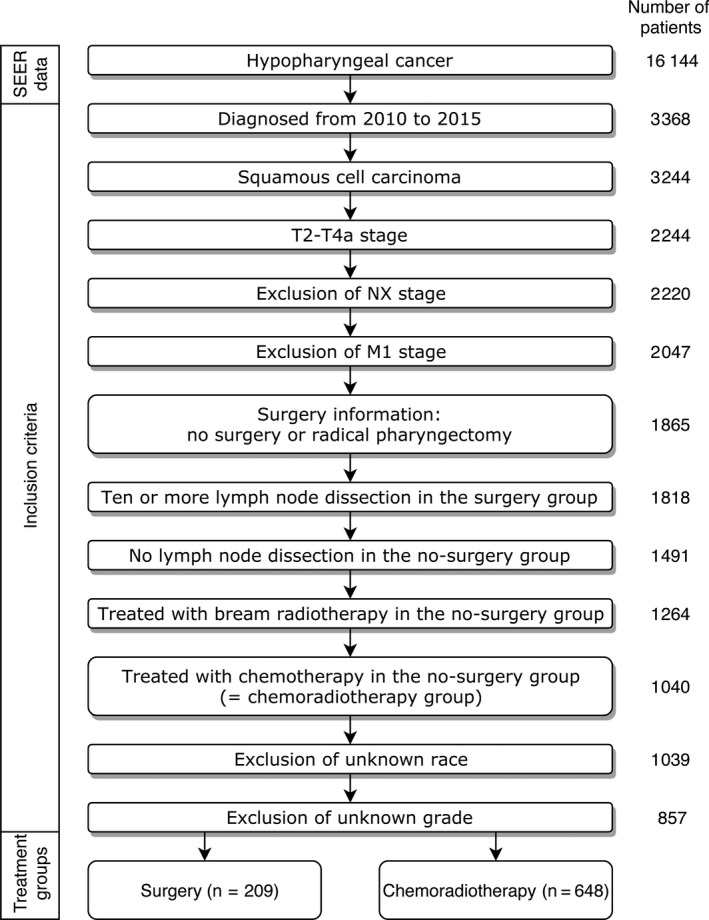
Flowchart of patient selection

SEER 18 registry were used to retrieve a list of patients. Inclusion criteria were as follows: (1) pathologically diagnosed hypopharynx squamous cell carcinoma between 2010 and 2015, (2) T2‐4aN0‐3M0 based on the 7th edition of the American Joint Committee on Cancer staging system, (3) having information of race, age, and tumor grade, (4) and treated with total pharyngectomy (code 32‐52) with 10 or more lymph nodes dissection (surgery group) or definitive external beam radiotherapy and chemotherapy without surgery or lymph node dissection (chemoradiotherapy group; Figure 1).

Surgery codes from 32 to 52 include total pharyngectomy, pharyngectomy with laryngectomy or removal of contiguous bone tissue, radical pharyngectomy which includes total mandibular resection with or without laryngectomy. All treatments (surgery, radiotherapy, and chemotherapy) were the first course of treatment at the time of diagnosis.

### 2.2 Statistical analysis

Characteristics of the surgery and chemoradiotherapy groups were compared using the Pearson’s chi‐squared test. Three‐year OS rates of the surgery and chemoradiotherapy groups were calculated by the Kaplan‐Meier survival estimate. Univariate analyses were performed to evaluate prognostic significances of clinicopathologic variables on the OS rates using the Kaplan‐Meier method followed by a log‐rank test. Variables which were significantly prognostic in the univariate analysis, or significantly different between the surgery and chemoradiotherapy groups, or considered clinically important were incorporated in a multivariate analysis using the Cox’s proportional hazard model. Two‐sided *P*‐value <0.05 were considered as statistically significant. The R software ver 3.5.1 (https://www.r-project.org) was used for all analysis. Graphs were created using the “survminer” R package.

## 3 RESULTS

### 3.1 Patient characteristics

The characteristics of patients are summarized in Table 1. The number of the patients who were diagnosed with hypopharyngeal cancer from 2010 to 2015 was 16144. Finally, the number of the patients who were satisfied the inclusion criteria was 857 (Figure 1). Among them, 209 (24.4%) and 648 (75.6%) patients were included in the surgery and chemoradiotherapy groups, respectively, suggesting that definitive chemoradiotherapy were more chosen as a primary treatment for locally advanced hypopharyngeal cancer.

**Table 1:** Patient characteristics

Characteristics	All			T2‐T3			T4a		
	Surgery	Chemoradiotherapy	*P* value*	Surgery	Chemoradiotherapy	*P* value*	Surgery	Chemoradiotherapy	*P* value*
	(N = 209)	(N = 648)		(N = 98)	(N = 522)		(N = 111)	(N = 126)	
Age									
<65 y	100 (47.8%)	358 (55.2%)	0.074	38 (38.8%)	287 (55.0%)	0.005	62 (55.9%)	71 (56.3%)	1.000
≥65 y	109 (52.2%)	290 (44.8%)		60 (61.2%)	235 (45.0%)		49 (44.1%)	55 (43.7%)	
Race									
White	164 (78.5%)	508 (78.4%)	0.947	75 (76.5%)	413 (79.1%)	0.735	89 (80.2%)	95 (75.4%)	0.653
Black	30 (14.4%)	97 (15.0%)		15 (15.3%)	77 (14.8%)		15 (13.5%)	20 (15.9%)	
Others	15 (7.2%)	43 (6.6%)		8 (8.2%)	32 (6.1%)		7 (6.3%)	11 (8.7%)	
Sex									
Male	171 (81.8%)	535 (82.6%)	0.888	77 (78.6%)	425 (81.4%)	0.604	94 (84.7%)	110 (87.3%)	0.695
Female	38 (18.2%)	113 (17.4%)		21 (21.4%)	97 (18.6%)		17 (15.3%)	16 (12.7%)	
Subsite									
Pyriform sinus	116 (55.5%)	318 (49.1%)	0.187	45 (45.9%)	256 (49.0%)	0.736	71 (64.0%)	62 (49.2%)	0.070
Other sites	38 (18.2%)	152 (23.5%)		23 (23.5%)	126 (24.1%)		15 (13.5%)	26 (20.6%)	
Not otherwise specified	55 (26.3%)	178 (27.5%)		30 (30.6%)	140 (26.8%)		25 (22.5%)	38 (30.2%)	
Grade									
1‐2	113 (54.1%)	390 (60.2%)	0.139	57 (58.2%)	317 (60.7%)	0.716	56 (50.5%)	73 (57.9%)	0.306
3‐4	96 (45.9%)	258 (39.8%)		41 (41.8%)	205 (39.3%)		55 (49.5%)	53 (42.1%)	
T category									
T2	36 (17.2%)	282 (43.5%)	<0.001	36 (36.7%)	282 (54.0%)	0.002	0 (0.0%)	0 (0.0%)	NA
T3	62 (29.7%)	240 (37.0%)		62 (63.3%)	240 (46.0%)		0 (0.0%)	0 (0.0%)	
T4a	111 (53.1%)	126 (19.4%)		0 (0.0%)	0 (0.0%)		111 (100.0%)	126 (100.0%)	
N category									
N0	67 (32.1%)	181 (27.9%)	0.230	45 (45.9%)	146 (28.0%)	0.004	22 (19.8%)	35 (27.8%)	0.522
N1	33 (15.8%)	135 (20.8%)		14 (14.3%)	113 (21.6%)		19 (17.1%)	22 (17.5%)	
N2	106 (50.7%)	314 (48.5%)		38 (38.8%)	247 (47.3%)		68 (61.3%)	67 (53.2%)	
N3	3 (1.4%)	18 (2.8%)		1 (1.0%)	16 (3.1%)		2 (1.8%)	2 (1.6%)	
Insurance									
Insured	152 (72.7%)	475 (73.3%)	0.664	74 (75.5%)	394 (75.5%)	0.909	78 (70.3%)	81 (64.3%)	0.592
Medicaid	47 (22.5%)	133 (20.5%)		19 (19.4%)	96 (18.4%)		28 (25.2%)	37 (29.4%)	
Uninsured/unknown	10 (4.8%)	40 (6.2%)		5 (5.1%)	32 (6.1%)		5 (4.5%)	8 (6.3%)	
Marriage									
Married	91 (43.5%)	315 (48.6%)	0.231	48 (49.0%)	259 (49.6%)	0.995	43 (38.7%)	56 (44.4%)	0.449
Others/unknown	118 (56.5%)	333 (51.4%)		50 (51.0%)	263 (50.4%)		68 (61.3%)	70 (55.6%)	
No. of examined lymph nodes									
0	0 (0.0%)	648 (100%)	<0.001	0 (0.0%)	522 (100%)	<0.001	0 (0.0%)	126 (100%)	<0.001
10≤	209 (100%)	0 (0.0%)		98 (100%)	0 (0.0%)		111 (100%)	0 (0.0%)	
Radiotherapy									
No	89 (42.6%)	0 (0.0%)	<0.001	56 (57.1%)	0 (0.0%)	<0.001	33 (29.7%)	0 (0.0%)	<0.001
Yes	120 (57.4%)	648 (100.0%)		42 (42.9%)	522 (100.0%)		78 (70.3%)	126 (100.0%)	
Chemotherapy									
No	121 (57.9%)	0 (0.0%)	<0.001	70 (71.4%)	0 (0.0%)	<0.001	51 (45.9%)	0 (0.0%)	<0.001
Yes	88 (42.1%)	648 (100.0%)		28 (28.6%)	522 (100.0%)		60 (54.1%)	126 (100.0%)	
Radiotherapy + Chemotherapy									
No	125 (59.8%)	0 (0.0%)	<0.001	73 (74.5%)	0 (0.0%)	<0.001	52 (46.8%)	0 (0.0%)	<0.001
Yes	84 (40.2%)	648 (100.0%)		25 (25.5%)	522 (100.0%)		59 (53.2%)	126 (100.0%)	

* Pearson’s chi‐squared test.

Patients in the surgery group were older (*P* = 0.074) and had higher T stage (*P* < 0.001) than those in the chemoradiotherapy group. In the subgroup of T2‐3 categories, patients who underwent chemoradiotherapy had significantly high N stage (*P* = 0.004)—approximately half of the patients who received surgery were N0 category (45.9%). In the subgroup of T4a category, the proportion of pyriform sinus tumor among the subsites showed higher tendency in the surgery group (*P* = 0.070). Otherwise, there were no significant differences between the two treatment groups in the T4a category.

Among all patients in the surgery group, radiotherapy was performed in 120 (57.4%) patients and chemotherapy was performed in 88 (42.1%) patients, respectively. The number of patients in the surgery group who were treated with both radiotherapy and chemotherapy was 84 (40.2%).

### 3.2 Overall survival rate

**Figure 2:**
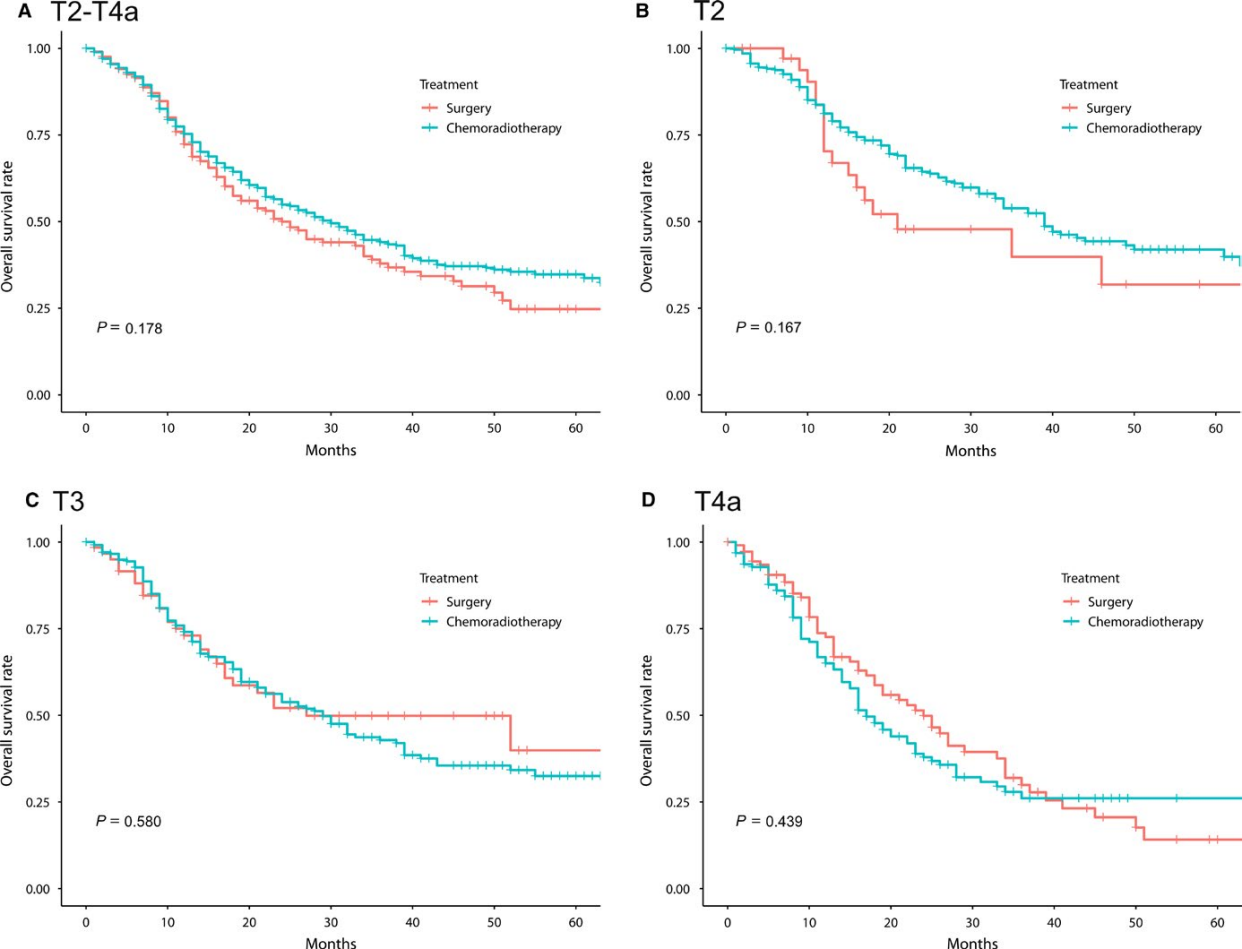
The Kaplan‐Meier overall survival estimate in locally advanced (T2‐T4a) hypopharyngeal cancer treated with surgery or definitive chemoradiotherapy. (A) T2‐T4a, (B) T2, (C) T3, and (D) T4a categories

Three‐year OS rate in the surgery and chemoradiotherapy groups was 37.9% and 44.1%, respectively (*P* = 0.178; Table 2; Figure 2A). No subgroup including T4a category showed statistically significant OS difference between the surgery and chemoradiotherapy group.

**Table 2:** Univariate analysis for overall survival rate in locally advanced hypopharyngeal cancer

Characteristics	Surgery		Chemoradiotherapy		*P* value*
	3‐y OS (%)	95% CI	3‐y OS (%)	95% CI	
All	37.9	30.5‐47.2	44.1	39.8‐48.9	0.178
Age					
<65 y	39.8	29.4‐54.1	49.2	43.5‐55.7	0.109
≥65 y	36.4	26.6‐49.8	37.5	31.3‐44.9	0.972
*P* value*	0.532		<0.001		<0.001
Race					
White	41.9	33.2‐52.9	45.9	41.0‐51.2	0.511
Black	28.1	14.3‐55.1	34.4	24.0‐49.3	0.188
Others	17.0	4.8‐60.0	42.7	29.3‐62.2	0.260
*P* value*	0.046		0.227		0.025
Sex					
Male	36.7	28.6‐47.2	44.4	39.7‐49.7	0.167
Female	43.5	28.1‐67.4	42.2	32.5‐54.8	0.827
*P* value*	0.722		0.716		0.872
Subsite					
Pyriform sinus	30.6	21.5‐43.5	46.0	40.0‐53.1	0.082
Other sites	47.5	32.2‐70.0	39.8	31.5‐50.5	0.878
Not otherwise specified	49.8	36.7‐67.6	44.2	36.5‐53.6	0.780
*P* value*	0.740		0.268		0.322
Grade					
1‐2	39.1	29.0‐52.8	46.8	41.4‐53.0	0.145
3‐4	36.6	26.5‐50.6	39.9	33.3‐47.9	0.770
*P* value*	0.926		0.167		0.194
T category					
T2	39.8	23.4‐67.7	53.9	47.3‐61.4	0.167
T3	49.9	37.7‐65.9	42.8	36.0‐50.9	0.580
T4a	29.9	20.5‐43.7	26.1	18.4‐37.0	0.439
*P* value*	0.200		<0.001		<0.001
N category					
N0	51.7	39.3‐67.9	46.0	38.2‐55.5	0.633
N1	43.2	25.7‐72.7	51.1	42.0‐62.2	0.895
N2	28.3	19.0‐41.9	41.9	35.9‐48.8	0.061
N3	33.3	6.7‐100.0	14.5	2.9‐72.0	0.355
*P* value*	0.216		<0.001		0.003
Insurance					
Insured	43.9	34.8‐55.3	47.7	42.7‐53.3	0.450
Medicaid	19.9	10.0‐39.4	32.9	24.4‐44.4	0.149
Uninsured/unknown	43.8	18.9‐100.0	36.6	22.5‐59.3	0.654
*P* value*	0.019		0.010		<0.001
Marriage					
Married	36.0	25.1‐51.6	52.1	45.9‐59.2	0.061
Others/unknown	35.4	25.9‐48.5	34.9	29.1‐41.9	0.536
*P* value*	0.629		<0.001		<0.001
No. of examined lymph nodes					
0	NA	NA	44.1	39.8‐48.9	NA
10≤	37.9	30.5‐47.2	NA	NA	NA
*P* value*	NA		NA		0.178
Radiotherapy					
No	36.1	26.1‐49.9	NA	NA	NA
Yes	39.1	29.2‐52.5	44.1	39.8‐48.9	0.921
*P* value*	0.081		NA		0.024
Chemotherapy					
No	37.2	28.0‐49.5	NA	NA	NA
Yes	39.0	27.9‐54.6	44.1	39.8‐48.9	0.914
*P* value*	0.203		NA		0.064
Radiotherapy + Chemotherapy					
No	36.7	27.6‐48.9	NA	NA	NA
Yes	40.0	28.6‐55.8	44.1	39.8‐48.9	0.951
*P* value*	0.139		NA		0.045

CI, confidence interval; NA, not applicable; OS, overall survival.

* Kaplan‐Meier survival estimate compared by a log‐rank test.

Since the T category distribution between the treatment groups differed, we also calculated the survival rate of each subgroup by the T category. The 3‐year OS rates for the T2‐3 patients in the surgery and chemoradiotherapy groups were 46.5% and 48.7% (*P* = 0.598) (39.8% vs. 53.9% for T2 [*P* = 0.167] and 49.9% vs. 42.8% for T3 [*P* = 0.580], respectively; Figure 2B,C). The 3‐year OS rate for the T4a patients was 29.9% and 26.1% in the surgery and chemoradiotherapy groups, respectively (*P* = 0.439; Figure 2D).

**Figure 3:**
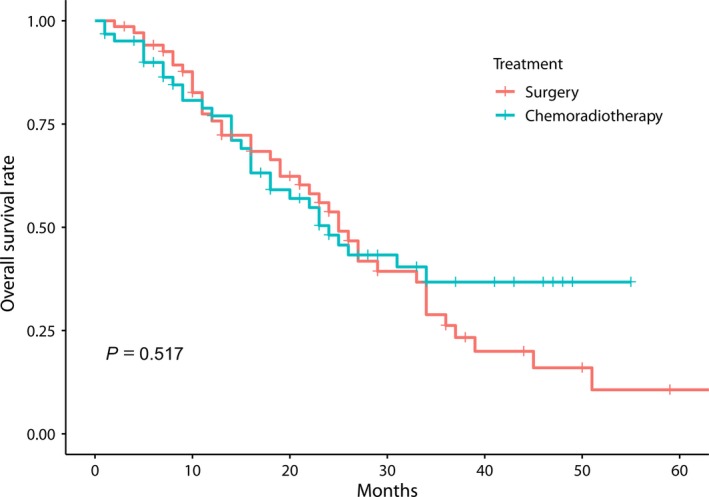
The Kaplan‐Meier overall survival estimate in T4a hypopharyngeal cancer at pyriform sinus treated with surgery or definitive chemoradiotherapy

As the distribution of subsites was different between the treatment groups in the T4a category, survival rate was separately calculated for pyriform sinus tumors in the T4a category. The 3‐year OS rates were 26.2% in the surgery group and 36.7% in the chemoradiotherapy groups (*P* = 0.517). Although there was no statistically significant difference in the survival rate between the treatment groups, the chemoradiotherapy group showed a stable survival rate after 36 months of survival (Figure 3).

On univariate analysis for the OS rate, patients who were aged ≥65 years (*P* = 0.001), non‐white race (*P* = 0.025), T4a category (*P* < 0.001), high N category (*P* = 0.003), Medicaid or uninsured (*P* < 0.001), and unmarried status (*P* < 0.001) were correlated with significantly dismal OS rates. Multivariate analysis was performed incorporating all variables—the treatment group (the surgery or chemoradiotherapy groups), age, race, sex, subsite, grade, T and N categories, insurance, and marital status—because all variables were statistically significant in the comparison between treatment group and/or the univariate analyses, or considered clinically important. On this multivariate analysis, the chemoradiotherapy group did not show a significantly adverse OS rate compared to the surgery group (hazard ratio [HR] 0.889, 95% confidence interval [CI] 0.699‐1.129, *P* = 0.334). Patients who were aged ≥65 years (*P* < 0.001), T4a (*P* < 0.001), N3 (*P* = 0.002), Medicaid (*P* = 0.002), and unmarried status (*P* = 0.002) were associated with poor OS rates after the multivariate analysis.

In subgroup analyses according to the T categories, the chemoradiotherapy group also showed similar OS rates compared to the surgery group. On multivariate analysis in the T2‐3 categories, HR of the chemoradiotherapy group compared to the surgery groups was 0.932 (95% CI 0.669‐1.297, *P* = 0.675). Even in the T4a category, a multivariate analysis incorporating all variables showed no significant OS difference between the surgery and chemoradiotherapy groups (HR of the chemoradiotherapy group 0.880, 95% CI 0.617‐1.256, *P* = 0.481). The subsites except pyriform sinus were a significantly adverse feature in the T4a category (HR 2.509, 95% CI 1.510‐4.170, *P* < 0.001; Table 3).

**Table 3:** Multivariate analysis for OS rate according to T category in locally advanced hypopharyngeal cancer

Characteristics	All			T2‐3			T4a		
	HR	95% CI	*P* value*	HR	95% CI	*P* value*	HR	95% CI	*P* value*
Treatment									
Surgery	Reference			Reference			Reference		
Chemoradiotherapy	0.889	0.699‐1.129	0.334	0.932	0.669‐1.297	0.675	0.880	0.617‐1.256	0.481
Age									
<65 y	Reference			Reference			Reference		
≥65 y	1.779	1.431‐2.210	<0.001	2.153	1.644‐2.820	<0.001	1.488	0.991‐2.235	0.056
Race									
White	Reference			Reference			Reference		
Black	1.216	0.928‐1.592	0.156	1.073	0.764‐1.505	0.686	1.370	0.844‐2.224	0.203
Others	1.16	0.791‐1.702	0.447	1.421	0.874‐2.310	0.157	0.952	0.504‐1.801	0.881
Sex									
Male	Reference			Reference			Reference		
Female	1.041	0.807‐1.342	0.759	1.031	0.762‐1.395	0.843	1.148	0.686‐1.919	0.600
Subsite									
Pyriform sinus	Reference			Reference			Reference		
Other sites	1.275	0.989‐1.644	0.061	1.013	0.751‐1.365	0.934	2.509	1.510‐4.170	<0.001
Not otherwise specified	1.003	0.795‐1.265	0.979	0.847	0.637‐1.125	0.252	1.252	0.827‐1.896	0.288
Grade									
1‐2	Reference			Reference			Reference		
3‐4	1.086	0.891‐1.324	0.415	1.302	1.021‐1.660	0.033	0.773	0.537‐1.112	0.165
T category									
T2	Reference			Reference					
T3	1.259	0.992‐1.599	0.059	1.261	0.990‐1.604	0.060	NA		
T4a	1.784	1.372‐2.320	<0.001	NA					
N category									
N0	Reference			Reference			Reference		
N1	0.999	0.736‐1.357	0.996	0.933	0.643‐1.355	0.715	1.121	0.637‐1.973	0.692
N2	1.259	0.997‐1.588	0.053	1.390	1.054‐1.833	0.020	1.008	0.653‐1.555	0.972
N3	2.472	1.409‐4.337	0.002	2.779	1.435‐5.381	0.002	2.942	0.923‐9.381	0.068
Insurance									
Insured	Reference			Reference			Reference		
Medicaid	1.507	1.167‐1.945	0.002	1.574	1.133‐2.187	0.007	1.468	0.944‐2.282	0.088
Uninsured/unknown	1.340	0.866‐2.073	0.189	1.606	0.941‐2.741	0.082	0.945	0.421‐2.124	0.892
Marriage									
Married	Reference			Reference			Reference		
Others/unknown	1.379	1.122‐1.694	0.002	1.362	1.058‐1.753	0.016	1.567	1.068‐2.299	0.022

CI, confidence interval; HR, hazard ratio; NA, not applicable; OS, overall survival.

* Cox proportional hazards model.

## 4 DISCUSSION

In comparison with definitive chemoradiotherapy and surgery for locally advanced hypopharyngeal cancer, similar OS rates were observed. Even in the T4a category, chemoradiotherapy did not decrease the OS rate, suggesting that definitive chemoradiotherapy may be a treatment option for the T4a hypopharyngeal cancer without sacrificing the OS rate.

Several studiesdemonstrated that 3‐year locoregional control rate of IMRT for locally advanced (Stage III‐IV) hypopharyngeal cancer is approximately 68%‐85%^15^, ^16^, ^17^, ^18^ and 5‐year local control rate was 53%‐63%,^18^, ^19^ and these results are similar with those of surgery.^19^, ^20^ The major pattern of failure of head and neck cancer patients who were treated with IMRT has become distant metastases rather than locoregional failures.^21^

Despite developed treatment modalities, however, the prognosis of locally advanced hypopharyngeal cancer is poor. With this perception, multidisciplinary decision making is mandatory to select optimal treatments. The key consideration for selection of treatment is the estimated survival, survival benefit from treatment, adverse effect after treatment, quality of life, and patient expectations.^22^

CategoryT4a (AJCC 7th) denotes the invasion of thyroid or cricoid cartilage, hyoid bone, thyroid gland, or central compartment soft tissue. Among them, the involvement of thyroid or cricoid cartilage were considered as risk factors decreasing the organ‐preserving possibility after chemoradiotherapy.^7^, ^23^ On the contrary, the invasion of thyroid or cricoid cartilage might not always cause a decrease in laryngopharyngeal dysfunction.^23^, ^24^ If the involvement of thyroid or cricoid cartilage is minor, chemoradiotherapy may provide an opportunity to conserve laryngopharyngeal functions.^25^

On the other hand, some patients may not be appropriate for chemoradiotherapy. The patients who had a tumor at the posterior wall of hypopharynx or retropharyngeal node invasion, and who had initial swallowing dysfunctions have high risks of dysphagia after chemoradiotherapy.^26^, ^27^, ^28^

However, it is noticeable that majority of locally advanced hypopharyngeal cancer patients who received surgery also need additional radiotherapy or chemoradiotherapy,^14^ and surgery is also an invasive procedure. General condition which is needed for surgical treatment may not be acceptable for all patients. IMRT, image‐guided radiotherapy (IGRT), and adaptive radiotherapy can reduce toxicity and improve quality of life after treatment even in old age,^29^, ^30^, ^31^ indicating that definitive chemoradiotherapy might be as beneficial as surgery if the patients are selected carefully.

Onepopulation‐based study using the National Cancer Data Base (NCDB) from 1998 to 2011 demonstrated that the treatment outcome of chemoradiotherapy for hypopharyngeal cancer are comparable between surgery with chemoradiotherapy and surgery with radiotherapy.^14^ In this NCDB study, T4a category was 29.2%, and subgroup analysis for locally advanced hypopharyngeal cancer was not performed. Therefore, the results from this NCDB study were not enough to confirm that chemoradiotherapy is comparable to surgery even in T4a category cancer.

Several studies insisted that (chemo)radiotherapy for T4a hypopharyngeal cancer showed a poor survival rate compared to surgery.^2^, ^5^, ^6^ On the other hand, a multi‐institutional study which performed a matched‐pair analysis between surgery and chemoradiotherapy between 2006 and 2008 showed no significant differences in survival or local control rates between the two treatment groups even in T4a category patients.^32^

We analyzed 791 of hypopharyngeal cancer patients diagnosed from 2004 to 2009 in the SEER database (T2‐4aN0‐3M0, AJCC 6th), resulting in the OS rate of T4a cancer patients who were treated with surgery was significantly superior to that of patients received chemoradiotherapy (data now shown). Therefore, the non‐inferiority of chemoradiotherapy compared to the surgery in T4a category might be an emerging result in this contemporary period.

One recent review study demonstrated that surgery and chemoradiotherapy showed similar survivorship in advanced hypopharyngeal carcinoma after reviewing two randomized trials and 11 observational studies^33^—in these included studies, considerable number of patients were T4 category, and especially the studies that covered the period of 2010s did not show a significant difference in OS between surgery and chemoradiotherapy.^20^, ^25^, ^33^, ^34^

Larynx preservation rate after chemoradiotherapy using IMRT for locally advanced hypopharyngeal cancer is reported as 89%‐96% at 2 years and 60% at 5 years.^12^, ^17^, ^18^ Therefore, if IMRT is performed for patients who were carefully selected based on the possibility of organ preservation, a considerable number of patients may be able to expect larynx preservation.

Several trials for hypopharyngeal cancer have started to study effective treatments for this disease. Neoadjuvant chemotherapy (NCT01312350), BKM120 (NCT02113878), and WEE1 Inhibitor (NCT03028766) with cisplatin‐based chemoradiotherapy, adaptive radiotherapy (NCT03096808), and upfront neck dissection with definitive chemoradiotherapy (NCT02918955) are evaluated. Also, effectiveness of laser therapy for mucositis induced by a chemoradiotherapy (NCT01772706) and swallowing rehabilitation on quality of life after radiotherapy (NCT02892487) are also investigated.

The limitation of this study mainly comes from the retrospective nature. All patients were real clinical data, and majority of the patients would be treated based on the multidisciplinary decisions. Even though T4a category patients are included in the same category, the severity of tumor might be more aggressive in the surgery group considering a low possibility of preserving the laryngopharyngeal function. Conversely, patients whose medical condition were not appropriate for surgical treatment may be treated with chemoradiotherapy.

Population database, but relatively small patient number owing to the rarity of the disease as well as the confined study period and short follow‐up period can be another limitation. However, because of the poor survival rate especially for T4a hypopharyngeal cancer, the follow‐up period may be sufficient to reach statistical significance if there is a significant difference.

Information of comorbidity, locoregional control rate, and radiotherapy regimen (total dose, fractionation, and fraction size), and radiotherapy modality (conventional or IMRT) were also not available. IMRT has been a major modality of radiotherapy for head and neck cancer.^11^, ^35^ Therefore, most of the patients in our study are supposed to be treated with IMRT.

All treatments in the SEER database are the first course of treatment after diagnosis. Induction chemotherapy and definitive concurrent chemotherapy could not be divided. However, based on the historical outcomes demonstrating no significant difference between the induction and concurrent regimens,^14^, ^36^, ^37^ the chemoradiotherapy group was considered as one integrated treatment group (“non‐surgical treatment” group).

In conclusion, definitive chemoradiotherapy for locally advanced hypopharyngeal cancer including T4a category showed a non‐inferior OS rate compared to surgery. For patients with T4a category cancer with high possibility of preserving the laryngopharyngeal function or with inoperable condition, chemoradiotherapy may be a promising alternative treatment.

## CONFLICT OF INTEREST

None.
